# ﻿Endophytic *Colletotrichum* (Sordariomycetes, Glomerellaceae) species associated with *Citrusgrandis* cv. “Tomentosa” in China

**DOI:** 10.3897/mycokeys.95.87121

**Published:** 2023-02-23

**Authors:** Jia-Wei Liu, Ishara S. Manawasinghe, Xuan-Ni Liao, Jin Mao, Zhang-Yong Dong, Ruvishika S. Jayawardena, Dhanushka N. Wanasinghe, Yong-Xin Shu, Mei Luo

**Affiliations:** 1 Innovative Institute for Plant Health/ Key laboratory of Fruit and Vegetable Green Prevention and Control in South-China, Ministry of Agricul-ture and Rural Affairs, Zhongkai University of Agriculture and Engineering, Guangzhou 510225, China; 2 Center of Excellence in Fungal Research, Mae Fah Luang University, Chiang Rai, Thailand; 3 School of Science, Mae Fah Luang University, Chiang Rai, Thailand; 4 Center for Mountain Futures, Kunming Institute of Botany, Chinese Academy of Sciences, Kunming, Honghe 654400, China

**Keywords:** Chinese traditional medicinal plants, new ascomycete, phylogeny, six new host records, taxonomy, two new species

## Abstract

*Colletotrichum* species are well-known plant pathogens, saprobes, endophytes, human pathogens and entomopathogens. However, little is known about *Colletotrichum* as endophytes of plants and cultivars including *Citrusgrandis* cv. “Tomentosa”. In the present study, 12 endophytic *Colletotrichum* isolates were obtained from this host in Huazhou, Guangdong Province (China) in 2019. Based on morphology and combined multigene phylogeny [nuclear ribosomal internal transcribed spacer (ITS), glyceraldehyde-3-phosphate dehydrogenase (*gapdh*), chitin synthase 1 (*chs-1*), histone H3 (*his3*) actin (*act*), beta-tubulin (β-*tubulin*) and glutamine synthetase (*gs*)], six *Colletotrichum* species were identified, including two new species, namely *Colletotrichumguangdongense* and *C.tomentosae*. *Colletotrichumasianum*, *C.plurivorum*, *C.siamense* and *C.tainanense* are identified as being the first reports on *C.grandis* cv. “Tomentosa” worldwide. This study is the first comprehensive study on endophytic *Colletotrichum* species on *C.grandis* cv. “Tomentosa” in China.

## ﻿Introduction

*Citrusgrandis* cv. “Tomentosa” is an important traditional medicinal plant which contains essential oils, flavonoids and polysaccharides. In traditional Chinese medicine, *Citrusgrandis* cv. “Tomentosa” has been used for treatments due to its anti-inflammatory effect (Zhao et al. 2017). It has also been used in the treatment of coughs, asthma, food stagnation, vomiting and other symptoms (Peng et al. 2019). Current research on *C.grandis* cv. “Tomentosa” is still focused on medicinal components, with a relatively long timescale needed to accumulate the effective ingredient. It is likely that the endophytic community living inside the host affects the metabolites of the plant. Dai et al. (2017) found that nine species of *Taxus* endophytic fungi could produce paclitaxel. Hasan et al. (2022) found endophytic fungi, *Penicilliumcrustosum* from *Annonamuricata* L. has anti-cancer activity against HeLa cells. Therefore, it is necessary to study the effects of the endophytic community associated with these traditional medicinal plants. The findings of this research can help in finding potential new natural medicines and form the basis for subsequent screening of strains.

*Colletotrichum*Corda (1831), belongs to Glomerellaceae (Sordariomycetes), which comprises plant pathogens, endophytes and saprobes on a wide range of hosts (Christy et al. 2020; Jayawardena et al. 2021). They are one of the most often isolated endophytic fungal groups encompassing a wide range of hosts. These endophytic *Colletotrichum* species have some advantages to the host, such as providing disease resistance, drought tolerance and promoting growth of the host (Hacquard et al. 2016; Dini-Andreote 2020). Endophytic species can also change their lifestyle and become pathogenic (Photita et al. 2004). Liu et al. (2022) accepted 280 *Colletotrichum* species, from which 23 species have been identified from *Citrus* spp. Therefore, studying diversity and clarifying taxonomic affinities of isolates can answer a range of important ecological and evolutionary questions. Although there have been several studies on *Colletotrichum* species associated with *Citrus* (Damm et al. 2012; Huang et al. 2013; Guarnaccia et al. 2017), there is still imprecise identification of endophytes of *Colletotrichum* species on *C.grandis* cv. “Tomentosa”.

Species delineation of *Colletotrichum* is challenging because there are few distinctive morphological characters available (Bhunjun et al. 2021). *Colletotrichum* is characterised as an intricate genus with 16 species complexes and 15 singleton species (Liu et al. 2022). Although host specificity was the most used character for identification in early studies, current taxonomic classifications and species delineations are based on morphology alongside multi-locus phylogeny (Bhunjun et al. 2021; Jayawardena et al. 2021; Liu et al. 2022). Phylogenetic analyses of *Colletotrichum* have been based on ITS, *gapdh*, *chs-1*, *act* and β-*tubulin* and multi-loci phylogeny. However, some complexes that cannot be distinguished by five loci required additional loci for identification (Bhunjun et al. 2021; Jayawardena et al. 2021; Liu et al. 2022). Therefore, the selection of gene combinations depends on the species complex (Jayawardena et al. 2021).

The objectives of this study were to isolate and identify the dominant endophytic *Colletotrichum* species associated with healthy *C.grandis* cv. “Tomentosa” in Huazhou, Guangdong, China. Morphology, molecular phylogeny and recombination analysis were used for the species characterisation. This resulted in two new species and six new host records. Detailed descriptions and coloured illustrations have been given for the novel taxa identified.

## ﻿Materials and methods

### ﻿Sample collection and isolation

Healthy leaves and twigs of *Citrusgrandis* cv. “Tomentosa” were randomly collected from a *Citrus* orchard in Huazhou, Guangdong Province, China (21°66'N, 110°63'E). A total of 20 trees were randomly selected for the collection. Ten samples were collected from the upper, middle and lower parts of each plant. Asymptomatic samples were packed into zip-lock bags in a foam box with ice and were then brought to the plant pathology laboratory of Zhongkai University of Agriculture and Engineering where they were preserved at 4 °C before processing. Isolation was undertaken within 48 h after collection, following the procedure by Dong et al. (2021).

Endophytic fungi were isolated following the methods described by da Silva et al. (2020). The samples were initially washed with running tap water followed by sterile water. The leaves were cut into 3 mm × 3 mm segments, while the twigs were cut into 3 mm long pieces. Each piece was then surface sterilised by being dipped sequentially into 75% ethanol for 30 s, 2.5% NaClO (sodium hypochlorite) for 30–60 s (leaves for 30 s, twigs for 60 s), before being rinsed three times with sterilised water. They were then dried on sterilised filter paper. The cuttings were then placed on potato dextrose agar (PDA: 200 g potato, 20 g dextrose, 20 g agar per 1 litre of water). Plates were incubated at 25 °C with 12 h of dark and 12 h of fluorescent light. Pure cultures were cultured on PDA for 7 to 14 days at 25 °C. All the pure cultures obtained in this study were deposited in the Culture Collection of Zhongkai University of Agriculture and Engineering (**ZHKUCC**). The living cultures (ex-type) of new species identified in this study were deposited in the Culture Collection of the Chinese Academy of Sciences (CGMCC, *C.guangdongense* for the holotype with CGMCC 3.24127 and *C.tomentosae* with CGMCC 3.24128). Herbarium materials as dry cultures of novel species were deposited in the Herbarium of Zhongkai University of Agriculture and Engineering (**ZHKU**). The strain numbers belonging to all isolates (from ZHKUCC 21-0095 to 21-0106 and 22-041 to 22-0042) for this study are presented in Appendix 1.

### ﻿Morphological studies

For macro- and micro-morphological characterisation, 5 mm diameter agar plugs were cut from all the actively growing pure cultures on PDA and were then transferred on to new PDA. The colony diameter was measured daily for 5–9 d to determine the growth rate (mm/day) on the PDA at 25 °C under 12 h of dark and 12 h of fluorescent light. Appressoria formation was observed following Johnston and Jones (1997) and Cai et al. (2009). The cultures were incubated for 2–4 weeks and morphological characters (appressoria, ascomata, asci, ascospores, conidiophores and conidia) were observed. Macro-morphological characters were photographed using a SteREO Discovery.V20 (Zeiss, Germany) stereomicroscope. Fruiting bodies were cut into thin sections by a CM1860 freezing sliding microtome (LEICA, Germany). Digital images were captured with an Eclipse 80i photographic microscope (Nikon, Japan). Measurements were taken using NIS Elements BR 3.2 (Nikon, Japan). The mean values were calculated with their standard deviations (SDs).

### ﻿DNA extraction, PCR amplification and sequencing

Total genomic DNA was extracted from mycelium grown on PDA and incubated for approx. seven days at 25 °C using the CTAB method (Sun et al. 2009). The ITS region was amplified and sequenced. The resulting sequences were subjected to BLASTn searches in GenBank (https://blast.ncbi.nlm.nih.gov) to identify them to the genus level. Once the BLAST results had confirmed isolates as being *Colletotrichum* species, an additional six gene regions, namely *gapdh*, *chs-1*, *his3*, *act*, β-*tubulin* and *gs*, were amplified and sequenced. The PCR conditions for each primer pair are given below (Table 1). The amplicons were observed on 1% agarose electrophoresis gel and positive amplicons were sequenced by Tianyi Huiyuan Biotechnology Co., Ltd., Guangzhou, China. The initial sequence quality was checked using BioEdit v. 7.25 (Hall 2006). A total of 66 sequences generated in this study were submitted to GenBank (Appendix 1).

**Table 1.** Gene regions, respective primer pairs and PCR protocols used in the study.

Gene	Primer pair	Optimised PCR protocols	References
ITS	ITS1	94 °C: 5 min (94 °C: 30 s, 53 °C: 30 s, 72 °C: 1 min) × 32 cycles, 72 °C: 10 min	White et al. (1990)
	ITS4		
*gapdh*	GDF	94 °C: 5 min (94 °C: 30 s, 60 °C: 30 s, 72 °C: 1 min) × 32 cycles, 72 °C: 10 min	Guerber et al. (2003)
	GDR		
*chs-1*	CHS-79F	94 °C: 5 min (94 °C: 30 s, 49 °C: 30 s, 72 °C: 1 min) × 32 cycles, 72 °C: 10 min	Carbone and Kohn (1999)
	CHS-345R		
*his3*	CYLH3F	94 °C: 5 min (94 °C: 30 s, 53 °C: 30 s, 72 °C: 1 min) × 32 cycles, 72 °C: 10 min	Crous et al. (2004)
	CYLH3R		
*act*	ACT-512F	94 °C: 5 min (94 °C: 30 s, 54 °C: 30 s, 72 °C: 1 min) × 32 cycles, 72 °C: 10 min	Carbone and Kohn (1999)
	ACT-783R		
*β-tubulin*	Bt2a	94 °C: 5 min (94 °C: 30 s, 58 °C: 30 s, 72 °C: 1 min) × 32 cycles, 72 °C: 10 min	Glass and Donaldson (1995)
	Bt2b		
*gs*	GSF1	94 °C: 5 min (94 °C: 30 s, 60 °C: 60 s, 72 °C: 1 min) × 35 cycles, 72 °C: 30 min	Guerber et al. (2003)
	GSR1		

### ﻿Phylogenetic analysis

For the phylogenetic analysis, reference sequences for *Colletotrichum* species and related taxa were obtained from NCBI GenBank (Appendix 1). Each locus was aligned together with the sequences obtained in the present study using MAFFT (https://www.ebi.ac.uk/Tools/msa/mafft/) (Katoh et al. 2019). Alignments were checked and manually adjusted where necessary with BioEdit v. 7.25 (Hall 2006). Alignment results were automatically trimmed using the Trimal tool in PhyloSuite (v.1.2.1) (Zhang et al. 2020). Phylogenetic analyses were conducted according to Maximum Likelihood (ML) in RAxML (Silvestro and Michalak 2010), maximum parsimony (MP) in PAUP (v.4.0) (Swofford 2002) and Bayesian analyses (BP) in MrBayes (v. 3.1.2) (Ronquist and Huelsenbeck 2003). The final analyses of the *Colletotrichumgloeosporioides* complex were made using the concatenated dataset of *act*, *chs-1*, *gapdh*, ITS, β-*tubulin* and *gs*, following Liu et al. (2022). The other two complexes: *Colletotrichumorchidearum* complex and *Colletotrichummagnum* complex were analysed using *act*, *chs-1*, *gapdh*, *his3*, ITS and β-*tubulin*, following Liu et al. (2022).

In the MP analysis, ambiguous regions were excluded and gaps were treated as missing data. Tree stability was evaluated with 1,000 bootstrap replications. Zero-length branches were collapsed and all the parsimonious trees were saved. Tree parameters: tree length (TL), consistency index (CI), retention index (RI), relative consistency index (RC) and homoplasy index (HI) were calculated. Kishino-Hasegawa tests (KHT) were conducted to evaluate the differences between the trees inferred as being under different optimality criteria (Kishino and Hasegawa 1989). MrModelTest v. 2.3 (Nylander 2004) was used to determine the evolutionary models for each locus to be used in Bayesian and Maximum Likelihood analyses. The Maximum Likelihood analyses were conducted using RAxML-HPC2 on XSEDE (8.2.8) (Stamatakis 2014) in the CIPRES Science Gateway platform (Miller et al. 2010). The GTR + I + G evolutionary model was employed with 1,000 non-parametric bootstrapping iterations. Bayesian analysis was performed in MrBayes v. 3.1.2 (Ronquist and Huelsenbeck 2003). Posterior probabilities (PPs) were determined using Markov Chain Monte Carlo sampling (MCMC). Six simultaneous Markov chains were run for 10^8^ generations, with sampling the trees at each 1000^th^ generation. From the 10,000 trees obtained, the first 2,500 representing the burn-in phase were discarded. The remaining 7,500 trees were then used to calculate the posterior probabilities (BPs) in a majority rule consensus tree. Taxonomic novelties were submitted to the FacesofFungi database (Jayasiri et al. 2015) and Index Fungorum (http://www.indexfungorum.org). The final sequence alignments generated in this study were submitted to TreeBASE (http://www.treebase.org) under the submission ID 29668.

### ﻿Pairwise homoplasy index (PHI) analysis

Recombination analyses were conducted to provide evidence for genetic distances for two new species identified, based on the phylogenetic analyses. The pairwise homoplasy index (Φw) (Bruen et al. 2006) was calculated in SplitsTree (version 4.1.4.4) using Kimura’s two-parameter (K2P) models for low genetic distance datasets. The standard deviation of split frequencies in the PHI test results (Φw) < 0.05 indicates significant recombination within the dataset.

## ﻿Results

In total, 12 endophytic *Colletotrichum* strains were obtained: seven from leaves and five from twigs. Based on the initial species identification undertaken through BLASTn searches, taxa isolated in this study belonged to three species complexes, namely the *C.gloeosporioides*, *C.magnum* and *C.orchidearum* complexes.

### ﻿*Colletotrichumgloeosporioides* complex

**Figure 1.**
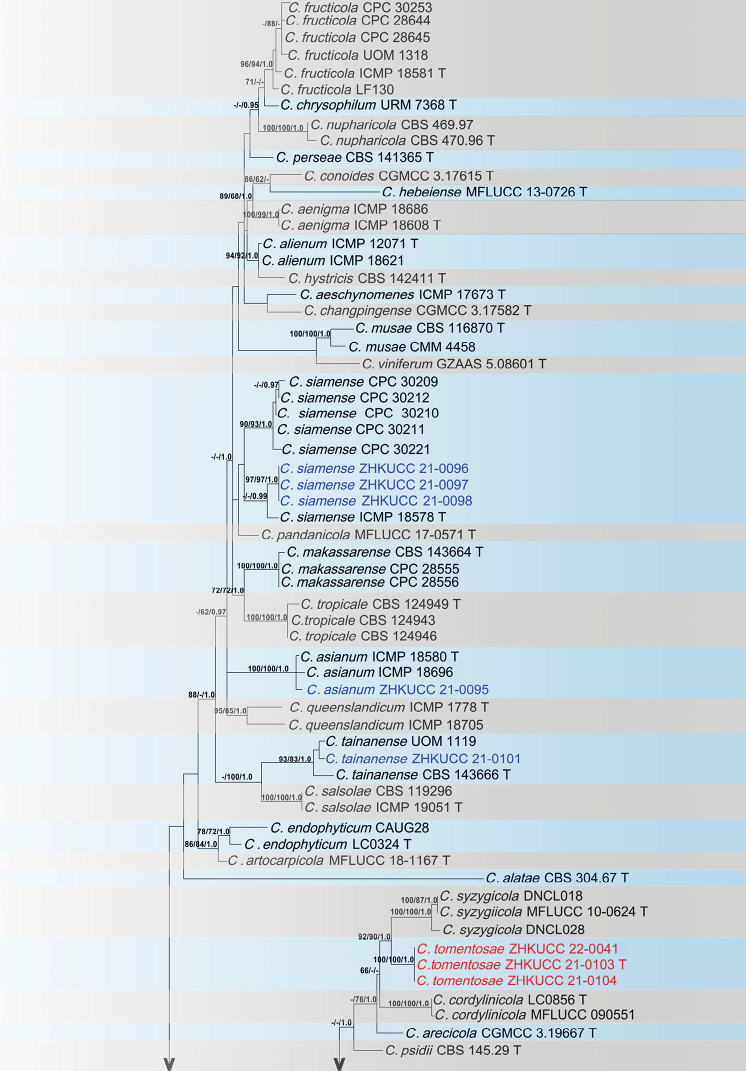
The most parsimonious tree of the *gloeosporioides* complex developed using combined *act*, *chs-1*, *gapdh*, ITS, β-*tubulin* and *gs* sequences. *Colletotrichumboninense* and *C.hippeastri* were used as outgroup taxa. Bootstrap values equal to or greater than 60% in MP and ML and BP equal to or greater than 0.95 are shown as MP/ML/BP above the respective node. The isolates belonging to the current study are given in blue for known species and new species are shown in red. Ex-type strains are noted with T.

**Figure 2.**
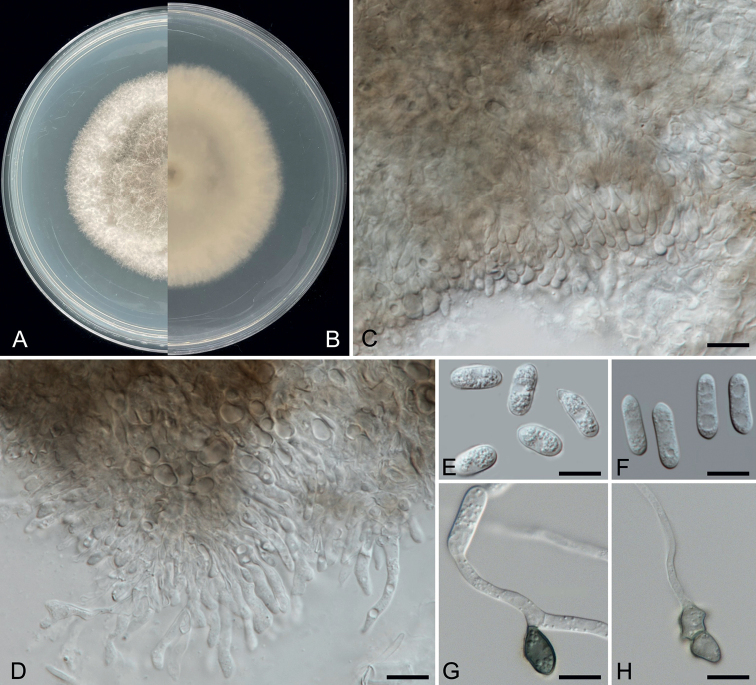
*Colletotrichumtomentosae* (ZHKUCC 21-0103, holotype) **A, B** upper and reverse side of cultures on PDA seven days after inoculation **C, D** conidiophores with developing conidia **E, F** conidia **G, H** appressoria. Scale bars: 10 μm (**C–H**).

In the present study, eight *Colletotrichum* isolates were initially recognised as belonging to the *C.gloeosporioides* complex. Phylogenetic analyses of a combined *act* (1–281), *chs-1* (282–573), *gapdh* (574–850), ITS (851–1384), β-*tubulin* (1385–1846) and *gs* (1847–2616) sequence alignment were conducted using 89 *Colletotrichum* strains. *Colletotrichumboninense* (ICMP 17904) and *C.hippeastri* (ICMP 17920) were used as outgroup taxa. The best-scoring MP tree is shown in Fig. 1. The dataset comprised 2,616 characters with 1,757 constant characters, 370 parsimony-informative and 489 parsimony-uninformative characters. The maximum number of trees generated was 1,000 and the most parsimonious trees had a length of 1,492 steps (CI = 0.707, RI = 0.848, RC = 0.600, HI = 0.293). The final ML tree topology was in line with the MP and BP trees. The best-scoring ML tree has a final likelihood value of −12,639.274168. The matrix consisted of 1,060 distinct alignment patterns, with 15.26% undetermined characters or gaps. For the Bayesian Inference, the TPM2uf+G model was selected for *act*, TIM1ef+G for *chs-1*, HKY+I for *gapdh*, TrNef+I+G for ITS, TIM3ef+G for β-*tubulin* and TVM+G for *gs.* In the phylogenetic analysis, three isolates (ZHKUCC 21-0103, ZHKUCC 21-0104 and ZHKUCC 22-0041) from this study developed a sister clade from other known species. The new species of *C.tomentosae* showed a close relationship to *C.syzygicola* (MFLUCC 10-0624) with 92% ML, 90% MP and 1.00 BP support. Three strains (ZHKUCC 21-0096, ZHKUCC 21-0097 and ZHKUCC 21-0098) from this study cluster together with *C.siamense* (ICMP 18578) with 0.99 BP support in the multi-locus phylogenetic tree. The strain ZHKUCC 21-0095 was clustered with *C.asianum* (ICMP 18580) with 100% ML, 100% MP and 1.00 BP in the phylogenetic tree. A single strain (ZHKUCC 21-0101) belongs to *C.tainanense* (CBS 143666) with 93% ML, 83% MP and 1.00 BP support. The PHI value indicates that there is no significant evidence for recombination amongst the species used in this analysis (p = 1.0) (Fig. 2). Based on this, we identified these isolates as novel *Colletotrichum* species. Species descriptions and illustrations of the new species, identified from the *C.gloeosporioides* complex, are presented below.

### ﻿Taxonomy

#### ﻿ Colletotrichum asianum

Taxon classificationFungiGlomerellalesGlomerellaceae

Prihast., L. Cai & K.D. Hyde, Fungal Diversity 39: 96 (2009)

5C82DBB3-CFC0-54A4-8EC7-466A475C0433

Index Fungorum Number: IF515408

Facesoffungi Number: FoF10689

##### Material examined.

China, Guangdong Province, Huazhou, isolated from healthy twigs of *Citrusgrandis* cv. “Tomentosa”, May 2019, Y.X. Shu, (dried culture ZHKU 21-0084); living culture ZHKUCC 21-095.

##### Notes.

The single isolate (ZHKUCC 21-0095) obtained in this study clustered with the *Colletotrichumasianum* ex-type strain (ICMP: 1850) with 100% ML, 100% MP and 1.0 BP values (Fig. 1). Morphologically, the isolate obtained in this study is similar to those in the original description of *C.asianum* (Prihastuti et al. 2009). This is the first report of *C.asianum* on *C.grandis* cv. “Tomentosa”.

#### ﻿ Colletotrichum siamense

Taxon classificationFungiGlomerellalesGlomerellaceae

Prihast., L. Cai & K.D. Hyde, Fungal Diversity 39: 98 (2009)

75590CA3-0B17-5518-A480-F26DF7E96487

Index Fungorum Number: IF515410

Facesoffungi Number: FoF03599

##### Material examined.

China, Guangdong Province, Huazhou, isolated from healthy leaf of *Citrusgrandis* cv. “Tomentosa”, May 2019, Y.X. Shu, (dried culture ZHKU 21-0085); living cultures ZHKUCC 21-0096, ZHKUCC 21-0097, ZHKUCC 21-0098).

##### Notes.

Three isolates obtained in this study (ZHKUCC 21-0096–100) clustered with the ex-type strain of *Colletotrichumsiamense* (ICMP: 18578) with 67% MP and 0.99 BP values (Fig. 1). Morphologically, the isolate obtained in this study is similar to those in the original description of *C.siamense* (Prihastuti et al. 2009). This is the first report of *C.siamense* on *C.grandis* cv. “Tomentosa”.

#### ﻿ Colletotrichum tainanense

Taxon classificationFungiGlomerellalesGlomerellaceae

de Silva, Crous & P.W.J. Taylor, IMA Fungus 10(1): 23 (2019)

9E6713DB-E25E-5111-A950-1835450F1E0C

Index Fungorum Number: IF827692

Facesoffungi Number: FoF10690

##### Material examined.

China, Guangdong Province, Huazhou, isolated from healthy leaf of *Citrusgrandis* cv. “Tomentosa”, May 2019, Y.X. Shu, (dried culture ZHKU 21-0086); living culture ZHKUCC 21-0101.

##### Notes.

A single isolate obtained in this study (ZHKUCC 21-0101) clustered with the *Colletotrichumtainanense* (CBS 143666) ex-type strain with 93% ML, 83% MP bootstrap and 1.0 BP values (Fig. 1). Morphologically, the isolate obtained in this study is similar to those in the original description of *C.tainanense* (de Silva et al. 2019). To our knowledge, this is the first report of *C.tainanense* on *C.grandis* cv. “Tomentosa”.

#### ﻿ Colletotrichum tomentosae

Taxon classificationFungiGlomerellalesGlomerellaceae

J.W. Liu, Manawas. & M. Luo sp. nov.

57D630D6-F8DD-5470-8F0F-08F809AB3BCE

Index Fungorum Number: IF559482

Facesoffungi Number: FoF10692

Fig. 2

##### Etymology.

The epithet refers to the cultivar of the host plant – *Citrusgrandis* cv. “Tomentosa”.

##### Holotype.

ZHKUCC 21-0103.

##### Description.

Endophytic in *C.grandis* cv. “Tomentosa” leaf. ***Sexual morph***: not observed. ***Asexual morph***: Conidiophores 20–40 × 3–5 μm (x− = 29.8 ± 5.5 × 3.7 ± 0.6 μm, n = 30), hyaline, cylindrical, 1–3-celled, unbranched or branched at the base. Conidia 10–20 × 3–6 μm (x− = 12.5 ± 1.6 × 4.4 ± 0.6 μm, n = 50), 1–2-guttulate, aseptate, straight, hyaline, smooth-walled, middle part cylindrical both ends obtuse, middle part occasionally shrinkage or bulging. Appressoria 5–15 × 5–10 μm (x− = 10 ± 1.8 × 7 ± 1.5 μm, n = 50) solitary or in loose groups, light brown to medium brown, Ellipsoidal to subcircular or irregular-shaped.

##### Cultural characteristics.

Colonies on PDA reach 70 mm diam. in seven days, with 10–11 mm/day (x− = 10 mm, n = 6) growth rate. Colonies flat with entire margin, floccose cottony, surface grey in the centre with glaucous margin. Reverse buff in the centre with off-white margin.

##### Material examined.

China, Guangdong Province, Huazhou, isolated from a healthy leaf of *Citrusgrandis* cv. “Tomentosa”, May 2019, Y.X. Shu, (dried cultures ZHKU 21-0088 ***holotype***); ex-type culture ZHKUCC 21-0103 (= CGMCC 3.24128), ex-isotype ZHKUCC 21-0104, ZHKUCC 22-0041).

##### Notes.

**Figure 3.**
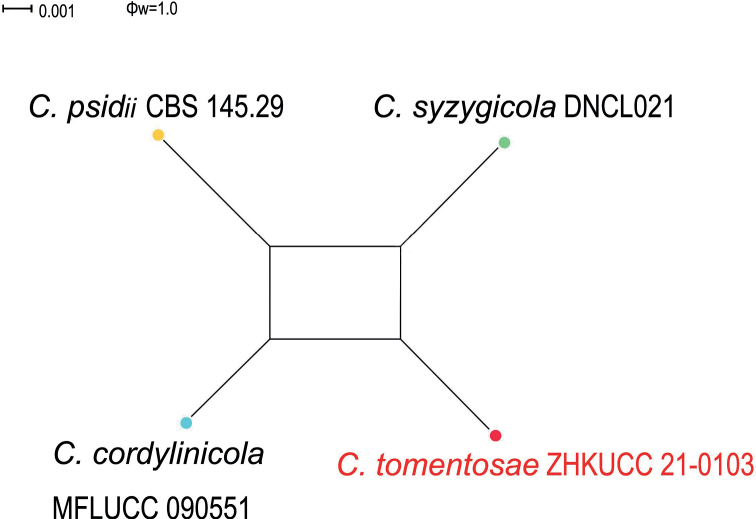
PHI analysis of combined ITS, *gapdh*, *chs-1*, *act* and β-*tubulin* sequence data. PHI test result (Φw) < 0.05 indicates significant recombination within the dataset.

In the phylogenetic analysis of combined six genes, *Colletotrichumtomentosae* formed an independent clade (Fig. 1). This species is phylogenetically distinct from *C.syzygicola*. Morphologically, appressoria developed by *C.syzygicola* (DNCL021; Udayanga et al. (2013)) are longer than *C.tomentosae* (5–15 × 18–24 μm vs. 18–24 μm). *Colletotrichumtomentosae* has longer conidiophores (20–40 × 3–5 vs. 12–16 × 4–5 μm). This species can be distinguished from *C.syzygicola* by 32 nucleotide differences (1/511 in the ITS region, 2/229 in the *gapdh* region, 7/242 in the *act* region and 22/906 in the *gs* region). The PHI test revealed no significant evidence for a recombination (p = 1.0) event amongst *C.syzygicola* and its closely-related taxa (Fig. 3). Therefore, we have described this fungus as a novel species.

### ﻿*Colletotrichumorchidearum* complex

**Figure 4.**
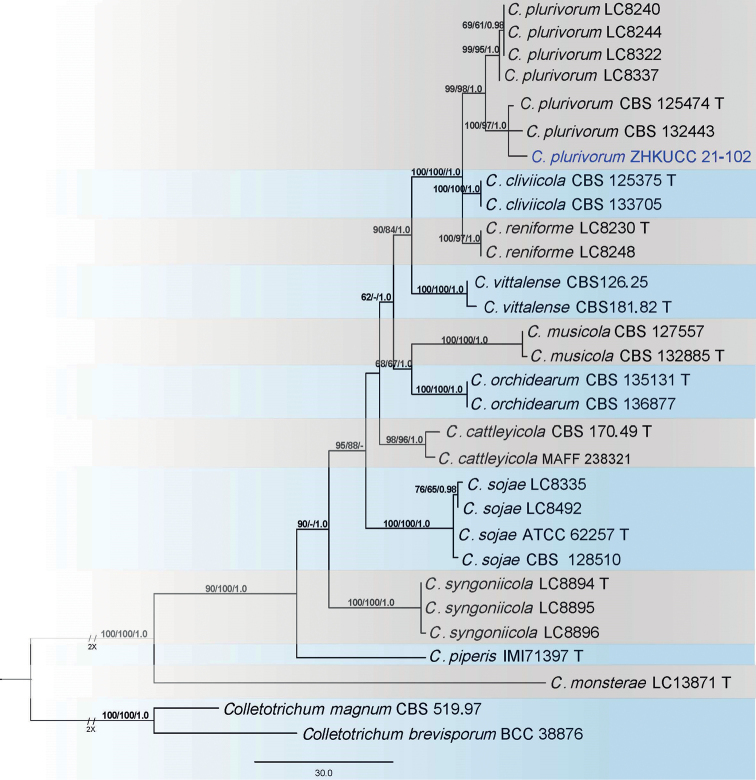
The most parsimonious tree for *Colletotrichumorchidearum* complex using a combined *act*, *chs-1*, *gapdh*, *his3*, ITS, and β-*tubulin* sequences. The tree is rooted to *Colletotrichumbrevisporum* and *C.magnum*. Bootstrap support values equal to or greater than 60% in MP and ML and BP equal to or greater than 0.95 are shown as MP/ML/BP above the respective nodes. The isolates belonging to the current study is given in blue. Ex-type strains are noted with T.

In the present study, a single isolate was recognised as belonging to the *Colletotrichumorchidearum* complex. The phylogenetic analysis of a combined ITS, *gapdh*, *chs-1*, *his3*, *act* and β-*tubulin* sequence alignment was constructed using 30 *Colletotrichum* strains. *Colletotrichummagnum* (CBS 519.97) and *C.brevisporum* (BCC 38876) were used as the outgroup. The best scoring MP tree is presented in Fig. 4. The dataset comprised 2,422 characters with 2,055 constant characters and 242 parsimony-informative and 125 parsimony-uninformative characters. The maximum number of trees generated was 1,000 and the most parsimonious trees had a length of 475 steps (CI = 0.874, RI = 0.904, RC = 0.790, HI = 0.126). The final ML tree topology was similar to the MP and BP trees. The best-scoring ML tree with a final likelihood value of – 6,065.417493 is shown in Fig. 4. The matrix comprised 479 distinct alignment patterns, with 10.74% of undetermined characters or gaps. The estimated base frequencies were as follows: A = 0.214401, C = 0.319513, G = 0.254583, T = 0.211503; substitution rates AC = 0.9523776, AG = 3.421321, AT = 0.568275, CG = 0.738898, CT = 6.093168, GT = 1.000000; gamma distribution shape parameter a = 0.814817. For the Bayesian Inference, the TPM1uf+I model was selected for *act*, GTR+I+G for *chs-1*, HKY+I for *gapdh*, TIM2+G for *his3*, TIM1+I for ITS and HKY+G for β-*tubulin*. In the phylogenetic analysis, isolates from this study clustered together with *C.plurivorum*. The species description and illustration are given below.

#### ﻿ Colletotrichum plurivorum

Taxon classificationFungiGlomerellalesGlomerellaceae

Damm, Alizadeh & Toy. Sato, Studies in Mycology 92: 31 (2019)

D5DD5537-5639-565B-9521-9490D3BD34AC

Index Fungorum Number: IF824228

Facesoffungi Number: FoF10691

##### Material examined.

China, Guangdong Province, Huazhou, isolated from healthy leaf of *Citrusgrandis* cv. “Tomentosa”, May 2019, YX Shu, (dried culture ZHKU 21-0087), living culture ZHKUCC 21-0102.

##### Notes.

A single isolate (ZHKUCC 21-0102) obtained in this study clustered with the ex-type strain of *C.plurivorum* (CBS 125474) with 99% ML, 97% MP and 1.0 BP support values (Fig. 4). Morphologically, the isolate obtained in this study is similar to those in the original description of *C.plurivorum* (Damm et al. 2019). *Colletotrichumplurivorum* was first introduced by Damm et al. (2019) as a pathogen on *Capsicumannuum* fruits and subsequently, has been reported as pathogens causing anthracnose or leaf spot diseases (Farr and Rossman 2022). This is the first report of *C.plurivorum* as an endophyte on *Citrusgrandis* cv. “Tomentosa”.

### ﻿*Colletotrichummagnum* complex

**Figure 5.**
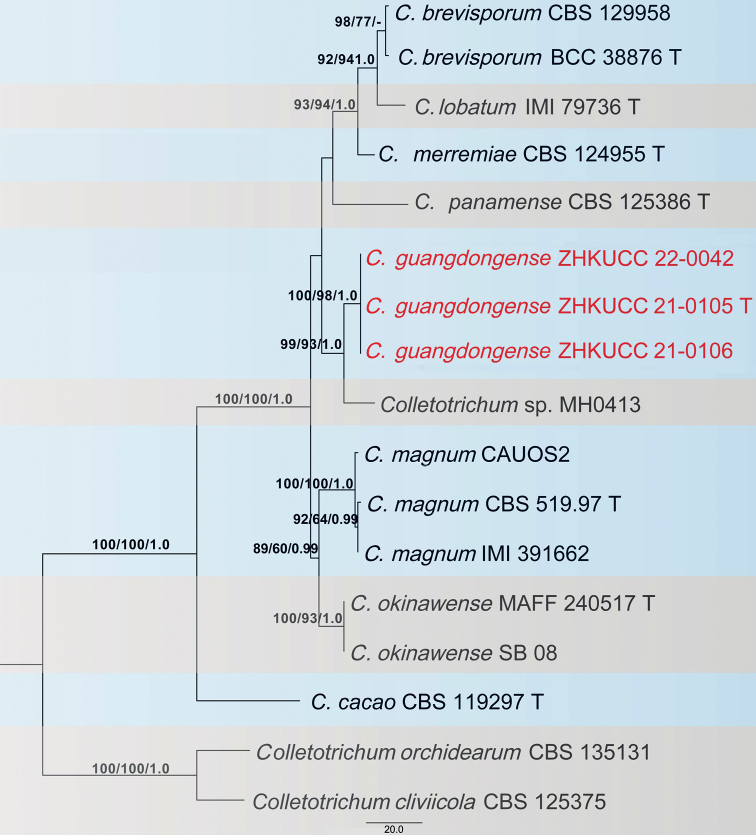
The most parsimonious tree of the *Colletotrichummagnum* complex using combined *act*, *chs-1*, *gapdh*, *his3*, ITS and β-*tubulin* sequences. *Colletotrichumcliviicola* and *C.orchidearum* were used as outgroup taxa. Bootstrap support values equal to or greater than 60% in MP and ML and BP equal to or greater than 0.95 are shown as MP/ML/BP above the respective nodes. The isolates of the novel taxon described in the current study are highlighted in red. Ex-type strains are noted with T.

**Figure 6.**
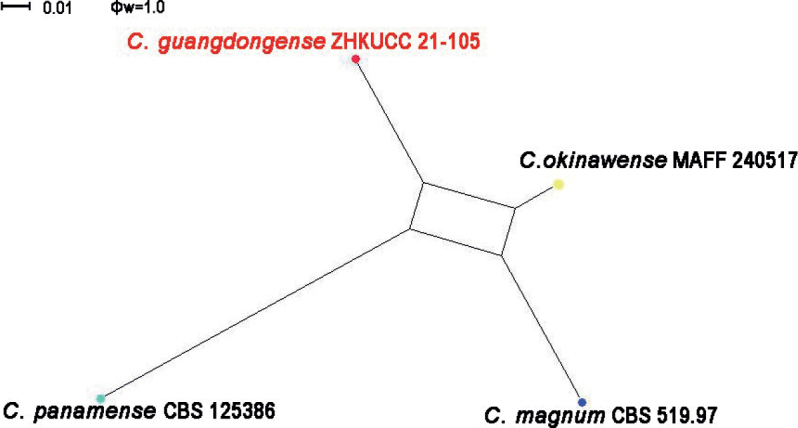
PHI analysis of combined *act*, *chs-1*, *gapdh*, *his3*, ITS and β-*tubulin* sequence data. A PHI test result (Φw) < 0.05 indicates significant recombination within the dataset.

Three of our isolates were initially recognised as belonging to the *Colletotrichummagnum* species complex. The phylogenetic analysis of combined *act*, *chs-1*, *gapdh*, *his3*, ITS and β-*tubulin* sequence alignment was conducted using 17 *Colletotrichum* strains. *Colletotrichumorchidearum* (CBS 135131) and *C.cliviicola* (CBS 125375) were used as outgroup taxa. The best-scoring MP tree is given in Fig. 5. The dataset consisted of 2,296 characters with 2,013 constant characters and 196 parsimony-informative and 87 parsimony-uninformative characters. The maximum number of trees generated was 1,000 and the most parsimonious trees had a length of 350 steps (CI = 0.883, RI = 0.882, RC = 0.779, HI = 0.117). The final ML tree topology was similar to the MP and BP trees. The best-scoring ML tree had a −5198.901460 final likelihood value. The ML matrix comprised 258 distinct alignment patterns, with 6.18% undetermined characters or gaps. For the Bayesian Inference, the HKY model was selected for *act*, TIM2ef+G for *chs-1*, HKY+G for *gapdh*, TrN+G for *his3*, TIM1+I for ITS and TIM1+G for β-*tubulin*. In the phylogenetic analysis, isolates from this study developed to show the presence of an independent clade with high bootstrap and BP support. To confirm that these isolates belonged to novel species, the PHI index was calculated. The PHI test revealed no significant evidence for recombination (p = 1.0) amongst the taxon from this study and its closely-related taxa (Fig. 6).

#### ﻿ Colletotrichum guangdongense

Taxon classificationFungiGlomerellalesGlomerellaceae

J.W. Liu, Manawas. & M. Luo sp. nov.

43281A0E-8EE6-52A9-AAD1-3D9B8B8BF57D

Index Fungorum Number: IF559483

Facesoffungi Number: FoF10693

Fig. 7

##### Etymology.

The epithet refers to the Guangdong Province where the fungus was collected.

##### Holotype.

ZHKUCC 21-0105

##### Description.

Isolated from a *Citrusgrandis* cv. “Tomentosa” twig. ***Sexual morph***: not observed. ***Asexual morph*.** Conidiomata formed directly on hyphae, conidial masses abundant, coral. Setae pale to dark brown, smooth-walled, straight or flexuous, 2–4-septate, 60–136 μm long, basal cell cylindrical, 3.5–4.8 μm diam., tip more or less acute. Conidiophores 20–70 × 3–7 μm (x− = 39.1 ± 10.7 × 4.7 ± 0.7 μm, n = 50), cylindrical, hyaline, smooth-walled, 1–4-celled, unbranched or branched at the base. Conidia 14–22 × 3–7 μm (x− = 18.2 ± 1.6 × 4.9 ± 0.5 μm, n = 50), straight, hyaline and smooth-walled. Appressoria 7–12 × 5–10 μm (x− = 10.2 ± 1.8 × 7.3 ± 0.9 μm, n = 50), single, medium brown, round, oval to irregular in outline.

**Figure 7.**
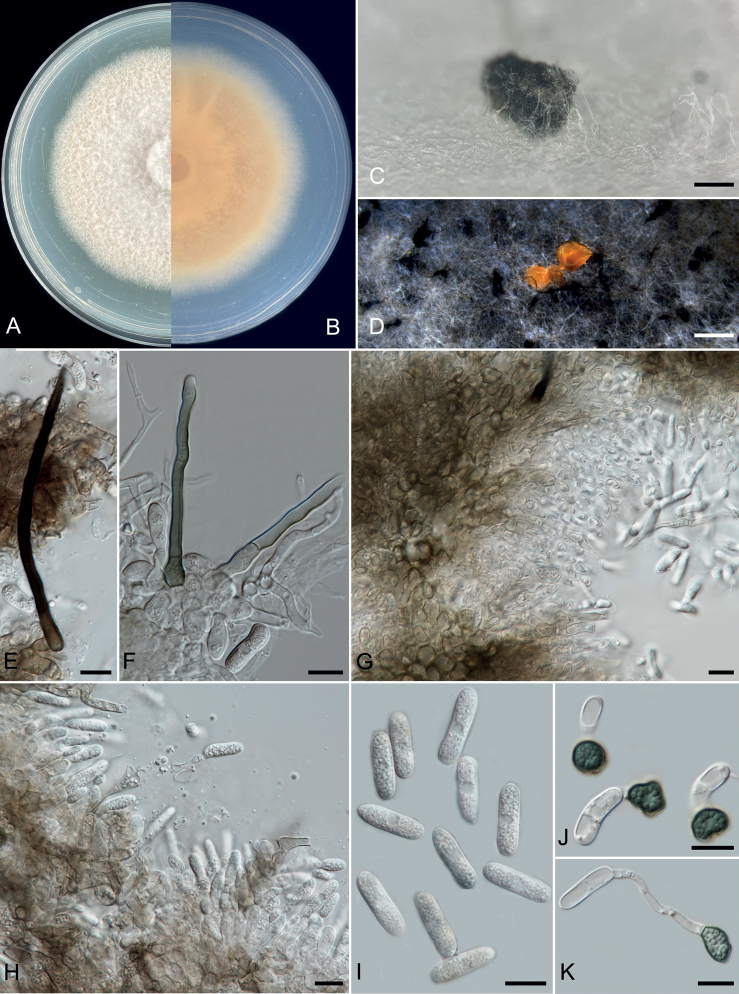
*Colletotrichumguangdongense* (ZHKUCC 21-0105, holotype) **A, B** upper and reverse sides of cultures on PDA seven days after inoculation **C, D** conidioma **E, F** setae **G, H** conidiophores **I** conidia **J, K** appressoria. Scale bars: 1 mm (**C, D**); 10 μm (**E–K**).

##### Cultural characteristics.

Colonies on PDA reach 65 mm diameter after seven days, with 8–11 mm/day (x− = 10 mm, n = 6) growth rate. Colonies circular, slightly raised, flat, with pale coral red to light pink margin. Reverse dark vermillion to light ivory. Colonies on SNA flat, with entire margin, glaucous, reverse buff. Sporulates after 14 d on SNA.

##### Material examined.

China, Guangdong Province, Huazhou, isolated from healthy twigs of *Citrusgrandis* cv. “Tomentosa”, May 2019, Y.X. Shu (dried cultures ZHKU 21-0089 ***holotype***); living cultures ZHKUCC 21-0105 (= CGMCC 3.24127) ex-type, ZHKUCC 21-0106 and ZHKUCC 22-0042 isotype).

##### Notes.

In the phylogenetic analysis of combined *act*, *chs-1*, *gapdh*, *his3*, ITS and β-*tubulin* sequences, three isolates (ZHKUCC 21-0105, ZHKUCC 21-0106 and ZHKUCC 22-0042) obtained in this study developed a sister clade to *Colletotrichum* sp. MH0413 with 89% ML bootstrap, 60% MP bootstrap and 1.00 BP (Fig. 5). *Colletotrichumguangdongense* is also closely related to *C.magnum* (CBS 519.97) and *C.panamense* (CBS 125386). It can be distinguished from *C.magnum* (CBS 519.97) by having smaller conidia (10–20 × 4–6 μm vs. 17–24 × 3.5–5 μm) and longer conidiophores (20–70 μm vs. 20 μm) (Damm et al. 2019). *Colletotrichumpanamense* (CBS 125386) has conidiophores shorter than *C.guangdongense* (30 μm vs. 20–70 μm). *Colletotrichumguangdongense* can be distinguished from *C.magnum* (CBS 519.97) also by 39 different nucleotides (4/538 in the ITS region, 9/204 in the *gapdh* region, 3/251 in the *chs-1* region, 9/235 *act*, 5/431 *tub2* and 9/403 *his3*) and from *C.panamense* (CBS 125386) by 39 different nucleotides (4/538 in the ITS region, 9/204 in the *gapdh* region, 3/251 in the *chs-1* region, 9/235 *act*, 2/431 *tub2* and 12/403 *his3*). The PHI test revealed no significant recombination event amongst *C.guangdongense* and its closely-related taxa (Fig. 6). Therefore, we have described this fungus as a novel species.

## ﻿Discussion

In the present study, endophytic *Colletotrichum* species were isolated from *Citrusgrandis* cv. “Tomentosa” in Guangdong Province, China. Guangdong Province has a mild subtropical monsoon climate with abundant rainfall and high average annual temperatures. Vigorous fruit trees provide suitable conditions for the colonisation of *Colletotrichum* species (Jayawardena et al. 2021). When the host is healthy, the endophyte has a symbiotic relationship with the host (Jayawardena et al. 2021). However, sometimes the interaction between the plant and the endophyte can switch from mutualistic to antagonistic or pathogenic (da Silva et al. 2020). Thus, the identification and characterisation of endophytic fungi are necessary. Based on the phylogenetic analysis using a combined seven loci (ITS, *gapdh*, *chs-1*, *act*, *his3*, *tub2* and *gs*), 12 isolates from this study were identified as being six distinct species within the three *Colletotrichum* species complexes (Figs 1, 4, 5). These results included two new species, namely *C.guangdongense*, *C.tomentosae* and three new host records for *C.asianum*, *C.plurivorum* and *C.tainanense*. *Colletotrichumsiamense* has also been identified and described as being associated with *Citrus*. The present study has re-affirmed that more than one *Colletotrichum* species can colonise a single host, which is consistent with the conclusion of Damm et al. (2012).

Species belonging to the *C.gloeosporioides* complex were often found as endophytes (Damm et al. 2012; Weir et al. 2012; Jayawardena et al. 2016). Here, we identified seven strains representing four species as endophytes from the *C.gloeosporioides* complex. *Colletotrichumsiamense* was previously reported as an epiphyte and an endophyte associated with coffee berries in northern Thailand (Prihastuti et al. 2009) and tea plants in China (Liu et al. 2015). *Colletotrichumsiamense* has also been reported as a pathogen of many plants (Liu et al. 2022). In the present study, this species was isolated from leaves. Liu et al. (2015) identified six species from symptomatic and asymptomatic leaf tissue, all of which belonged to the *C.gloeosporioides* species complex, namely *C.camelliae*, *C.fructicola*, *C.gloeosporioides*, *C.jiangxiense* and *C.siamense*, providing convincing evidence that these species could switch their lifestyle from endophytic to pathogenic. Therefore, further studies are necessary to understand the pathogenicity of these endophytic strains and the factors affecting these taxa becoming pathogenic on *Citrus*.

*Colletotrichum* species belonging to the *C.magnum* and *C.orchidearum* complexes were found on tropical or subtropical plants (Damm et al. 2019). It has been proposed that some of these species might be host- and region-specific (Damm et al. 2019). *Colletotrichumplurivorum* is widely distributed in several hosts and most of them are pathogens. This study is the first report of the species from *Citrus*. Here, we introduce a new taxon belonging to the *C.magnum* species complex. Whether it is host-specific or not needs further confirmation.

Endophytic fungal colonisation might vary in different tissues of the same plant (Taylor et al. 1999; Huang et al. 2015). Different fungal genera could have different tissue specificities and preferences. In the present study, endophytes were isolated from leaves and twigs. Additionally, there were higher numbers of *Colletotrichum* species from leaves in *Citrus* (Hakimeh et al. 2019) and some other plants like *Dendrobium* (Chen et al. 2011; Ma et al. 2018). Huang et al. (2015) and Dong et al. (2021) have observed that endophytic *Diaporthe* species are less abundant on leaves, whereas endophytic *Colletotrichum* species are abundantly isolated from the *Dendrobium* spp. leaves (Chen et al. 2011; Ma et al. 2018). These variations may be the result of differences in the tissue organisational structure, different nutrition contents of each tissue type or the lifestyle of each genus, locality or season (Zhou et al. 2014; Huang et al. 2015). To date, the reasons for these variations are not yet known.

Overall, in the present study, two novel endophytic *Colletotrichum* species have been described and illustrated. Our study is the first comprehensive study on endophytic *Colletotrichum* species associated with *Citrusgrandis* cv. “Tomentosa”. Moreover, our molecular data and novel species introduced in this study contribute to understanding the diversity and biology of the genus *Colletotrichum*. These results provide an important resource and basis for plant pathologists and fungal taxonomists. However, future studies are necessary to understand the lifestyle changes of the endophytic taxa towards the pathogenicity, as well as the effects of fungus-related medicinal properties of *Citrusgrandis* cv. “Tomentosa”.

## Supplementary Material

XML Treatment for Colletotrichum asianum

XML Treatment for Colletotrichum siamense

XML Treatment for Colletotrichum tainanense

XML Treatment for Colletotrichum tomentosae

XML Treatment for Colletotrichum plurivorum

XML Treatment for Colletotrichum guangdongense

## ﻿Appendix I

**Table A1.** Fungal isolates and sequences of molecular marker used in *Colletotrichum* phylogenetic analysis.

Species	Culture	Type	Genbank accession number						
			ITS	*gapdh*	*chs-1*	*his3*	*act*	*tub2*	*gs*
*C.gloeosporioides* complex									
*C.aenigma*	ICMP 18608	Holotype	JX010244	JX010044	JX009774	-	JX009443	JX010389	JX010078
	ICMP 18686		JX010243	JX009913	JX009789	-	JX009519	JX010390	JX010079
*C.aeschynomenes*	ICMP 17673	Holotype	JX010176	JX009930	JX009799	-	JX009483	JX010392	JX009799
*C.alatae*	ICMP 17919	Holotype	JX010190	JX009990	JX009837	-	JX009471	JX010383	JX010065
*C.alienum*	ICMP 12071	Holotype	JX010251	JX010028	JX009882	-	JX009572	JX010411	JX010101
	ICMP 18621		JX010246	JX009959	JX009755	-	JX009552	JX010386	JX010075
*C.aotearoa*	ICMP 18537	Holotype	JX010205	JX010005	JX009853	-	JX009854	JX010420	JX010113
*C.arecicola*	CGMCC 3.19667	Holotype	NR171191	-	-	-	-	-	-
*C.artocarpicola*	MFLUCC 181167	Holotype	MN415991	-	-	-	-	-	-
*C.asianum*	ICMP 18580	Holotype	JX010196	JX010053	JX009867	-	JX009584	JX010406	JX010096
	ICMP 18696		JX010192	JX009915	JX009753	-	JX009576	JX010384	JX010073
	ZHKUCC 21-0095		OL708418	OL855857	OL855867	-	OL855877	OL855883	-
*C.boninense*	ICMP 17904		JX010292	JX009905	JX009827	-	JX009583	-	-
*C.camelliae*	CGMCC3 14925	Holotype	KJ955081	KJ954782	MZ799255	-	KJ954363	KJ955230	KJ954932
*C.changpingense*	MFLUCC 150022	Holotype	KP683152	KP852469	KP852449	-	KP683093	KP852490	-
*C.chrysophilum*	CMM 4268	Holotype	KX094252	KX094183	KX094083	-	KX093982	KX094285	KX094204
*C.cigarro*	ICMP 18539	Holotype	JX010230	JX009966	-	-	JX009523	-	-
*C.clidemiae*	ICMP 18658	Holotype	JX010265	JX009989	JX009877	-	JX009537	JX010438	JX010129
*C.cobbittiense*	BRIP 66219	Holotype	NR_163538	MH094133	MH094135	-	MH094134	MH094137	-
*C.conoides*	CAUG17	Holotype	KP890168	KP890162	KP890156	-	KP890144	KP890174	-
*C.cordylinicola*	ICMP 18579	Holotype	JX010226	JX009975	JX009864	-	JX009586	JX010440	JX010122
	LC0856		HM470246	HM470240	-	-	HM470234	HM470249	HM470243
*C.endophytica*	CAUG28		KP145441	KP145413	KP145385	-	KP145329	KP145469	-
	MFLUCC 13-0418	Holotype	KC633854	KC832854	-	-	KF306258	-	-
*C.fructicola*	LF130		KJ955083	KJ954784	-	-	KJ954365	KJ955232	-
	CPC:28644		MH728811	MH707465	MH805851	-	MH781481	MH846564	-
	CPC:28645		MH728810	MH707466	MH805852	-	MH781482	MH846565	-
	CPC:30253		MH728817	MH707463	MH805846	-	MH781476	MH846559	-
	UOM 1138		MH728808	MH707468	MH805854	-	MH781484	MH846567	-
*C.fructicola*	ICMP 18581	Holotype	JX010165	JX010033	JX009866	-	JX009501	JX010405	JX010095
*C.fructivorum*	CBS 133125	Holotype	JX145145	MZ664047	MZ799259	-	MZ664126	JX145196	-
*C.gloeosporioides*	IMI 356878		JX010152	JX010056	JX009818	-	JX009531	JX010445	JX010085
*C.grevilleae*	CBS 132879	Holotype	KC297078	KC297010	KC296987	-	KC296941	KC297102	KC297033
*C.grossum*	CAUG7	Holotype	KP890165	KP890159	KP890153	-	KP890141	KP890171	-
*C.hebeiense*	MFLUCC 13-0726	Holotype	KF156863	KF377495	KF289008	-	KF377532	KF288975	-
*C.hederiicola*	MFLU 150689	Holotype	MN631384	-	MN635794	-	MN635795	-	-
*C.helleniense*	CPC:26844		KY856446	KY856270	KY856186	-	KY856019	KY856528	-
*C.henanense*	CGMCC 3.17354	Holotype	KJ955109	KJ954810	MZ799256	-	KM023257	KJ955257	KJ954960
*C.hippeastri*	ICMP 17920		JX010293	JX009932	JX009838	-	JX009485	-	-
*C.horii*	NBRC 7478	Holotype	GQ329690	GQ329681	JX009752	-	JX009438	JX010450	JN937000
*C.hystricis*	CBS 142411	Holotype	KY856450	KY856274	KY856190	-	KY856023	KY856532	-
*C.jiangxiense*	CGMCC 3.17363	Holotype	KJ955201	KJ954902	-	-	KJ954471	KJ955348	KJ955051
*C.kahawae*	ICMP 17816		JX010231	JX010012	JX009813	-	JX009452	JX010444	JX010130
*C.makassarense*	CPC:28612	Holotype	MH728812	MH728820	MH805850	-	MH781480	MH846563	MH748264
	CPC:28555		MH728816	MH728822	MH805847	-	MH781477	MH846560	MH748261
	CPC:28556		MH728815	MH728821	MH805848	-	MH781478	MH846561	MH748262
*C.musae*	CBS:116870	Holotype	JX010146	JX010050	JX009896	-	JX009433	HQ596280	JX010103
	CMM 4458		KX094249	KX094191	KX094080	-	KX093967	KX094292	KX094234
*C.nupharicola*	CBS 470.96	Holotype	JX010187	JX009972	JX009835	-	JX009437	JX010398	-
	CBS 469.96		JX010189	JX009936	JX009834	-	JX009486	JX010397	-
*C.pandanicola*	MFLUCC 170571	Holotype	MG646967	MG646934	MG646931	-	MG646938	MG646926	-
*C.perseae*	GA100	Holotype	KX620308	KX620242	-	-	KX620145	KX620341	KX620275
*C.proteae*	CBS 132882	Holotype	KC297079	KC297009	KC296986	-	KC296940	KC297101	KC297032
*C.pseudotheobromicola*	MFLUCC 181602	Holotype	MH817395	MH853675	MH853678	-	MH853681	MH853684	-
*C.psidii*	CBS 145.29	Holotype	JX010219	JX009967	JX009901	-	JX009515	JX010443	JX010133
*C.queenslandicum*	ICMP 1778	Holotype	JX010276	JX009934	JX009899	-	JX009447	JX010414	JX010104
	ICMP 18705		JX010185	JX010036	JX009890	-	JX009490	JX010412	JX010102
*C.rhexiae*	CBS 133134	Holotype	NR_144797	MZ664046	MZ799258	-	MZ664127	-	-
*C.salsolae*	ICMP 19051	Holotype	JX010242	JX009916	JX009863	-	JX009562	JX010403	JX010093
	CBS 119296		JX010241	JX009917	JX009791	-	JX009559	-	-
*C.siamense*	ICMP 18578	Holotype	JX010171	JX009924	JX009865	-	FJ907423	JX010404	JX010094
	CPC:30210		MH707472	MH707453	MH805835	-	MH781465	MH846548	MH748232
*C.siamense*	CPC:30211		MH707473	MH707454	MH805836	-	MH781466	MH846549	MH748233
	CPC:30212		MH707474	MH707455	MH805837	-	MH781467	MH846550	MH748234
	CPC:30221		MH707475	MH707456	MH805838	-	MH781468	MH846551	MH748235
	CPC:30209		MH707471	MH707452	MH805834	-	MH781464	MH846547	MH748231
	ZHKUCC 21-0096		OL708414	OL855849	OL855859	-	OL855869	OL855879	-
	ZHKUCC 21-0097		OL708424	OL855852	OL855862	-	OL855872	OL855881	-
	ZHKUCC 21-0098		OL708423	OL855851	OL855861	-	OL855871	OL855880	-
*C.syzygicola*	DNCL021	Holotype	KF242094	KF242156	-	-	KF157801	KF254880	KF242125
	DNCL028		KF242095	KF242157	-	-	KF157802	KF254881	KF242126
	DNCL018		KF242093	KF242155	-	-	KF157800	KF254879	KF242124
*C.tainanense*	CBS 143666	Holotype	MH728818	MH728823	MH805845	-	MH781475	MH846558	MH748259
	UOM 1119		MH728805	MH728819	MH805857	-	MH781487	MH846570	-
	ZHKUCC 21-0101		OL708421	OL855858	OL855868	-	OL855878	OL855884	-
*C.temperatum*	CBS 133122	Holotype	MH877532	MZ664045	MZ799254	-	MZ664125	-	-
*C.theobromicola*	CBS 124945	Holotype	JX010294	JX010006	JX009869	-	JX009444	JX010447	JX010139
*C.ti*	ICMP 4832	Holotype	JX010269	JX009952	JX009898	-	JX009520	JX010442	JX010123
	ICMP 5285		JX010267	JX009910	JX009897	-	JX009553	JX010441	JX010124
*C.tomentosae*	ZHKUCC 21-0103 CGMCC 3.24128 Dry culture: ZHKU 21-0088	Holotype	OL708422	OL855850	OL855860	-	OL855870	OL855887	ON315373
	ZHKUCC 21-0104		OL708419	OL855856	OL855866	-	OL855873	OL855888	ON315374
	ZHKUCC 22-0041		ON303476	ON315382	ON315376	-	ON315380	ON315378	ON315375
*C.tropicale*	CBS 124946		KC566806	KC566660	KC566373	-	KC566952	KC566228	-
	CBS 124943		JX010277	JX010014	JX009868	-	JX009570	-	-
	CBS 124949	Holotype	JX010264	JX010007	JX009870	-	JX009489	JX010407	JX010097
*C.viniferum*	GZAAS 5.08601	Holotype	JN412804	JN412798	-	-	JN412795	JN412813	-
*C.wuxiense*	CGMCC 3.17894	Holotype	KU251591	KU252045	KU251939	-	KU251672	KU252200	KU252101
*C.xanthorrhoeae*	BRIP 45094	Holotype	JX010261	JX009927	JX009823	-	JX009478	JX010448	JX010138
*C.yulongense*	CFCC 50818	Holotype	MH751507	MK108986	MH793605	-	MH777394	MK108987	MK108988
*C.magnum* complex									
*C.brevisporum*	BCC 38876	Holotype	JN050238	JN050227	MZ799287	MZ673841	JN050216	JN050244	-
	CBS 129958		MG600763	MG600823	MG600870	MG600909	MG600967	MG601030	-
*C.cacao*	CBS 119297	Holotype	MG600772	MG600832	MG600878	MG600916	MG600976	MG601039	-
*C.cliviicola*	CBS 125375		MG600733	MG600795	MG600850	MG600892	MG600939	MG601000	-
*C.guangdongense*	ZHKUCC 21-0105 CGMCC 3.24127 Dry culture: ZHKU 21-0089	Holotype	OL708415	OL855854	OL855864	ON315370	OL855875	OL855885	-
	ZHKUCC 21-0106		OL708420	OL855855	OL855865	ON315371	OL855876	OL855886	-
	ZHKUCC 22-0042		ON303474	ON315383	ON315377	ON315372	ON315381	ON315379	-
*C.lobatum*	IMI79736	Holotype	MG600828	MG600874	MG600912	MG600972	MG600972	MG601035	-
*C.magnum*	CBS519.97	Holotype	MG600769	MG600829	MG600875	MG600913	MG600973	MG601036	-
	IMI391662		MG600771	MG600831	MG600877	MG600915	MG600975	MG601038	-
	CAUOS2		MZ595839	MZ848400	OK236385	MZ673858	OK236387	MZ673960	-
*C.merremiae*	CBS124955	Holotype	MG600765	MG600825	MG600872	MG600910	MG600969	MG601032	-
*C.okinawense*	MAFF240517	Holotype	MG600767	MG600827	-	-	MG600971	MG601034	-
	SB 08		MK830706	MK820658	-	MK820660	MK820659	-	-
*C.orchidearum*	CBS135131		MG600738	MG600800	MG600855	MG600897	MG600944	MG601005	-
*C.panamense*	CBS125386	Holotype	MG600766	MG600826	MG600873	MG600911	MG600970	MG601033	-
*Colletotrichum* sp.	MH0413		MZ595871	MZ664109	MZ799289	MZ673891	MZ664169	MZ673990	-
*C.orchidearum* complex									
*C.brevisporum*	BCC 38876		JN050238	JN050227	MZ799287	MZ673841	JN050216	JN050244	-
*C.cattleyicola*	CBS 170.49	Holotype	MG600758	MG600819	MG600866	MG600905	MG600963	MG601025	-
	MAFF 238321		MG600759	-	-	-	-	MG601026	-
*C.cliviicola*	CBS 133705		MG600732	MG600794	MG600849	MG600891	MG600938	MG600999	-
	CBS 125375	Holotype	MG600733	MG600795	MG600850	MG600892	MG600939	MG601000	-
*C.magnum*	CBS519.97		MG600769	MG600829	MG600875	MG600913	MG600973	MG601036	-
*C.monsterae*	LC13871	Holotype	MZ595897	MZ664121	MZ799351	MZ673917	MZ664195	MZ674015	-
*C.musicola*	CBS132885	Holotype	MG600736	MG600798	MG600853	MG600895	MG600942	MG601003	-
	CBS127557		MG600737	MG600799	MG600854	MG600896	MG600943	MG601004	-
*C.orchidearum*	CBS135131	Holotype	MG600738	MG600800	MG600855	MG600897	MG600944	MG601005	-
	CBS136877		MG600739	MG600801	MG600856	MG600898	MG600945	MG601006	-
*C.piperis*	IMI71397	Holotype	MG600760	MG600820	MG600867	MG600906	MG600964	MG601027	-
*C.plurivorum*	CBS125474	Holotype	MG600718	MG600781	MG600841	MG600887	MG600925	MG600985	-
	CBS132443		MG600719	MG600782	MG600842	MG600888	MG600926	MG600986	-
	LC8240		MZ595848	MZ664113	MZ799291	MZ673868	MZ664146	MZ673969	-
	LC8244		MZ595849	MZ772868	MZ799292	MZ673869	MZ664147	MZ673970	-
	LC8322		MZ595853	MZ664114	MZ799293	MZ673873	MZ664151	MZ673974	-
	LC8337		MZ595855	MZ664115	MZ799294	MZ673875	MZ664153	MZ673976	-
	ZHKUCC 21-0102		OL708416	OL855874	OL855863	-	OL855853	OL855882	-
*C.reniforme*	LC8230	Holotype	MZ595847	MZ664110	MZ799290	MZ673867	MZ664145	MZ673968	-
	LC8248		MZ595850	MZ664111	MZ799295	MZ673870	MZ664148	MZ673971	-
*C.sojae*	ATCC62257	Holotype	MG600749	MG600810	MG600860	MG600899	MG600954	MG601016	-
	CBS128510		MG600751	MG600812	MG600862	MG600901	MG600956	MG601018	-
	LC8335		MZ595854	MZ664112	MZ799300	MZ673874	MZ664152	MZ673975	-
	LC8492		MZ595858	MZ664116	MZ799301	MZ673878	MZ664156	MZ673979	-
*C.syngoniicola*	LC8894	Holotype	MZ595863	MZ664117	MZ799296	MZ673883	MZ664161	MZ673982	-
	LC8895		MZ595864	MZ664118	MZ799297	MZ673884	MZ664162	MZ673983	-
	LC8896		MZ595865	MZ664119	MZ799298	MZ673885	MZ664163	MZ673984	-
*C.vittalense*	CBS126.25		MG600735	MG600797	MG600852	MG600894	MG600941	MG601002	-
	CBS181.82	Holotype	MG600734	MG600796	MG600851	MG600893	MG600940	MG601001	-
